# Clinical Heterogeneity in a Scandinavian *FMR1* Premutation Carrier Cohort and Basal Ganglia Atrophy in FXTAS

**DOI:** 10.1007/s12311-026-01968-6

**Published:** 2026-02-13

**Authors:** Sofia Berglund, Farouk Hashim, José Laffita-Mesa, Helena Malmgren, Britt-Marie Anderlid, Tobias Granberg, Henrik Sjöström, Per Svenningsson, Martin Paucar

**Affiliations:** 1 Department of Clinical Neuroscience, Karolinska Institutet, Stockholm, Sweden; 2 Department of Neuroradiology, Karolinska University Hospital, Stockholm, Sweden; 3 Department NVS, Division of Neurogeriatrics, Center for Alzheimer Research, Karolinska Institutet, Stockholm, Sweden; 4 Department of Molecular Medicine and Surgery, Karolinska Institutet, Stockholm, Sweden; 5 Department of Clinical Genetics and Genomics, Karolinska University Hospital, Stockholm, Sweden; 6 Center for Neurology, Academic Specialist Center, Stockholm, Sweden; 7 Department of Neurology, Karolinska University Hospital, Stockholm, Sweden

**Keywords:** FXTAS, *FMR1*, MRI neuroimaging, Ataxia

## Abstract

Pathological CGG expansions in the *FMR1* gene, encompassing premutations (55–200 repeats) and full range mutations (> 200 repeats), cause a spectrum of complex and incurable disorders. Premutation carriers (PMC), particularly men, face the risk of developing fragile X-associated tremor/ataxia syndrome (FXTAS). Other conditions associated with premutations in *FMR1* include both Fragile X-associated primary ovarian insufficiency (FXPOI), and Fragile X-associated neuropsychiatric disorders (FXAND). Hyperintensities in the middle cerebellar peduncle (MCP), known as the MCP sign, have been considered a radiological hallmark for FXTAS. *FMR1*-related disorders have not been characterized in Scandinavia. Here, we delineate the clinical spectrum of *FMR1* related disorders among PMC emphasizing neuroimaging abnormalities and assess the correlation between CGG repeat size and clinical parameters as well as neuroimaging abnormalities at a tertiary center in Sweden. In total 33 PMC were evaluated (19 women and 14 men) of which 21 received a *FMR1* related disorder (64%). As expected, penetrance for a *FMR1* related disorder was higher among men (11/14 [79%]) than in women (12/19, [63%]). We also found that hyperintensities in the splenium of corpus callosum (CCS sign) were more prevalent than the MCP sign, and volumetric analyses revealed atrophy in the basal ganglia, cerebellar grey and white matter among FXTAS patients. CGG expansion size correlated with motor scores but not with age of onset. Our findings indicate a high prevalence of *FMR1-*related disorders in this PMC cohort in which the CCS sign is more common than the MCP sign. Larger cohort studies are necessary to assess atrophy of the basal ganglia and its clinical correlations among *FMR1* PMC.

**Supplementary Information:**

The online version contains supplementary material available at 10.1007/s12311-026-01968-6.

## Introduction

Fragile X syndrome (FXS), caused by full CGG repeat expansions (> 200 repeats) in the fragile X messenger ribonucleoprotein 1 (*FMR1)* gene, is the most common form of hereditary intellectual function (IF) disability among boys [1]. Smaller CGG repeat expansions (55–200) in *FMR1*, called premutations, are associated with a wide spectrum of conditions that include Fragile X-associated tremor/ataxia syndrome (FXTAS) [2].

FXTAS is an incurable, late-onset neurodegenerative, and progressive syndrome that mainly affects male premutation carriers (PMC) [2–4]. Its penetrance increases with age affecting 75% of men older than 80 years of age and 8–16% of female PMC [2–4]. Intention tremor and cerebellar gait ataxia are the core motor manifestations of FXTAS along with variable presence of cognitive decline, parkinsonism, autonomic dysfunction, sensorimotor axonal polyneuropathy and psychiatric manifestations (e.g. anxiety, agitation, disinhibition and depression) [3, 5–10]. Longer repeat expansions are associated with both earlier age of onset and age of death [11]; likewise, some studies have found correlation with the repeat expansion size and severity of motor signs and degree of brain atrophy [12, 13], but other studies did not replicate this correlation [8]. The clinical presentation of FXTAS in women tends to be milder than in men [10, 14]. White matter abnormalities (WMA), in supratentorial and infratentorial regions, the splenium of corpus callosum (CCS sign), and increased signal intensity of the middle cerebellar peduncles (MCP sign) are the neuroimaging features of FXTAS [3]. Initially regarded as a hallmark for FXTAS [3], subsequent studies reported that the MCP sign was present only in 60% of men and 11% of women with FXTAS [15–17]. WMA in the CCS is as common as the MCP sign but has a higher sensitivity which has raised a call for revised diagnostic criteria [9, 18, 19].

Premutations in *FMR1* are also associated with Fragile X-associated primary ovarian insufficiency (FXPOI), and Fragile X-associated neuropsychiatric disorders (FXAND) [1]. FXPOI is defined as menopause due to hypogonadotropic hypogonadism before 40 years of age [20–22] and female PMC face a 20% risk of FXPOI [23–25]. FXAND, on the other hand, is proposed to include a spectrum of manifestations ranging from anxiety, depression, obsessive compulsive disorder, attention deficit hyperactivity disorder (ADHD), and even substance abuse [1]. The prevalence of *FMR1* PMC and in the general population is estimated to be 1 in 178 females and 1 in 400 males in the US [26, 27].

Here we describe a Swedish *FMR1* PMC cohort with emphasis on neuroimaging, and correlate CGG repeat size with clinical parameters and abnormalities on neuroimaging. We also performed an exploratory search for prevalence of *FMR1* premutations among healthy controls and in a large database.

## Patients and Methods

### Patients

This cross-sectional study, performed during the peak of the COVID19 pandemic (2020–2021), was approved by the Swedish Ethical Review Authority (The Regional Ethics Review Board in Stockholm). All patients, or their next of kin, provided oral and written consent. In total 33 PMC were included. All patients, but one, were evaluated at the Karolinska University Hospital, a tertiary center in Sweden (See supplementary document for details). Inclusion criteria were the presence of a premutation in the *FMR1* gene and age ≥ 18 years. Exclusion criteria were the presence of either a grey zone allele (45–54 CGG repeats) or a full *FMR1* mutation (> 200 CGG). The Jacquemont criteria were used to assign diagnosis as definite, probable and possible FXTAS [3]. Neurological features were assessed using the Inventory of Non-Ataxia Signs (INAS) and Scale for Assessment and Rating of Ataxia (SARA) with a cut-off value of 3 in SARA is compatible with ataxia. For cognition, the Montreal Cognitive Assessment (MoCA) was applied, MoCA ≤ 26 points was used as the cut-off for cognitive impairment. Psychiatric symptoms were evaluated with the Hospital Anxiety and Depression Scale (HADS). Since psychiatric symptoms are common in the general population, criteria are still to be well-defined, we assigned patients as affected by FXAND when they were affected by psychiatric conditions as defined in the Diagnostic and Statistical Manual of Mental Disorders, Fourth Edition (DSM-IV) criteria.

### Collection of Radiological and Pathological Parameters

Conventional structural brain exams with refined MRI algorithms were performed on 18 of the 33 *FMR1* PMC; 10 fulfilling FXTAS diagnostic criteria, and 8 PMC individuals not fulfilling FXTAS criteria. These scans were performed on 1.5 T and 3 T Siemens and GE scanners. A control group of 20 healthy volunteers were also recruited (See supplementary document). The control group underwent MR imaging using a Siemens Prisma(fit) 3 T scanner. Two neuroradiologists (FH and TG) evaluated for the presence of white matter changes, MCP sign, CCS sign, cortical and cerebellar atrophies.

Brain structures were automatically segmented using cNeuro cMRI (Combinostics, Tampere, Finland). Normalization was then performed using a method where the adjusted volumes were derived as follows: Volume adjusted = Volume raw - β * (HSF - HSFmean), where β is the slope of the regression line between the head size scale factor (HSF) and the respective brain structure volumes [28]. For this normalization, we used β-values and HSF mean derived from the control group (age and sex matched) and applied them to both the FXTAS/PMC and control groups.

### Genetic Analyses and Prevalence Estimates

The size of CGG nucleotide repeat expansions in the 33 PMC was determined at the Department of Genetics and Genomics at the Karolinska University Hospital. Briefly, analyses with PCR using primers flanking the CGG repeat in the *FMR1* gene were performed, followed by fragment analysis on an ABI3500 (Applied Biosystems). The analysis was performed on genomic DNA extracted from peripheral blood samples using the kit AmplideX^®^ PCR/CE *FMR1* Reagents (Asuragen) and analyzed using the software AmplideX^®^ PCR/CE FMR1 Reporter (Asuragen). The size of the expanded CGG repeat is determined with an accuracy of +/- 1–3 CGG, depending on the size of the premutation. A screening for premutations in DNA samples from anonymous blood donors from the Swedish Blood Donor Register was performed; for details see supplementary document.

### Statistical Analysis

Logistic regression analyses using SPSS were applied to assess the risk for FXTAS based on CGG repeat size, age, and sex; these same variables were used to predict cognitive decline/dementia and atrophy in the basal ganglia. The results are displayed as odds ratios (ORs) and 95% confidence intervals (CIs), with *p* values measuring the significance of the OR; all analyses were 2-tailed. Values for statistical tests are reported as mean ± SD, significance level was set at *p*<0.05.

For the statistical analyses of the MRI variables, SPSS (version 26.0, IBM Corp., Armonk, NY, USA) was used. Possible group differences in age were examined by one-way ANOVA and gender distribution between the groups was examined using Pearson’s chi-square test. Pearson’s chi-square test was also used to assess differences in the binary and ordinal MRI variables. Group differences in the volumetric MRI data were evaluated using ANCOVA with age as a covariate. In case of significant ANCOVA result, pairwise comparisons were then performed within the ANCOVA using the least significant difference (LSD). The significance level was set to *p* < 0.05.

## Results

### Clinical Features

In total 33 patients were evaluated, 19 women and 14 men. All but 4 patients were of Swedish origin- (88%), 28 have an *FMR1* related disorder (85%); and penetrance was higher among men (11/14[77%]) than in women (12/19[63%]) (Tables 1 and 2). Briefly, 14 patients meet the criteria for FXTAS (3 with definite, and 11 with probable FXTAS), 11 of them were men and 3 women (Table 2). Two of these women were sisters (Pts 9 and 11), both affected with an aggressive and lethal course of disease. Besides ataxia, one of them displayed signs of supranuclear palsy (PSP) which was confirmed by means of neuropathological assessment (Pt9) [29]. Her deceased sibling also had intranuclear inclusions typical for FXTAS but the staining for tau was normal (manuscript in preparation). All patients with FXTAS had a progressive disease, but missing data precluded evaluation of disease progression rate. Of the 19 female PMCs, three patients (16%) had FXPOI; 8 female and 2 male PMC had suffered from psychiatric symptoms that fall in the rage for what is considered part of the FXAND spectrum, psychiatric diagnosis and available HADS scores are presented in the supplementary document (see Table A1). One of these patients, diagnosed with FXTAS and PSP, suffered from major psychiatric symptoms predating motor onset (Pt 9) [29]. Mean CGG number for the entire cohort was 88.9 ± 20.1 repeats.

**Table 1:** Diagnoses in a PMC *FMR1* cohort at a tertiary center in Sweden. In total 33 patients were evaluated. *Two patients, both suffering from FXTAS, in one case also PSP, had an aggressive course of disease (Siblings assigned as Pts 9 and 11). Key: FXAND: fragile X-associated neuropsychiatric disorders; FXPOI: fragile X-associated primary ovarian insufficiency; FXTAS: fragile X-associated tremor/ataxia syndrome; NA: no applicable

Diagnosis (*n*)	Women (*n*)	Men (*n*)
FXTAS (14)	3	11
FXPOI (3)	3	NA
FXAND (10)	8*	2
Asymptomatic PMC (12)	9	3

**Table 2:** Demographic data of a *FMR1* permutation cohort at a tertiary center in Stockholm, Sweden, 29 patients were Swedish, other ethnic backgrounds are as follows: Pt 3 was Canadian, Pt 15 Finnish, Pt 22 Syrian and Pt 29 Chinese. In all the affected Dysautonomia consisted of variable degrees of urinary bladder affection and obstipation. Only one patient had had recurrent hypotonia (Pt 3) but this was attributed to severe heart failure. *Diagnosed as definite FXTAS, patients 9 and 11 (siblings) had neuropathology proven FXTAS, patient 9 had also PSP. ^α^Patients with parkinsonism and reduced binding to the dopamine transporter on PET, DaTSCAN was normal for patient 33. ** this patient presented with menopause at age 40. Widespread atrophy in this summary stands for affection to both supra- and infratentorial affection. ^In the right parietal lobe only. Key: BG: basal ganglia; CC: corpus callosum; cereb: Cerebellum; N: No; Y: Yes, park: NA: not applicable; park: Parkinsonism; psych: psychiatric. – Neuroimaging was not performed. The size of the CGG repeat has an accuracy of +/-1-3 CGG depending on the size of the premutation using a kit from Asuragen. Atrophy in BG, cerebellum and CC determined by volumetric analyses

Patients and parameters	Gender	Status/ diagnosis	Age at onset/ age at diagnosis/*Age at death* (years)	CGG repeats in expanded allele	CGG in the healthy allele of women	Ataxia/ SARA (latest score)	Tremor	Parkins.	Cognitive decline	Psych. features	Neuro-pathy/ Dysautonomia	MCP sign	WMA/ CCS sign	Atrophy in the BG/Another atrophy
Patient 1	M	FXTAS	65/68	112	NA	Y/NA	Y	Y	23/ Dementia	N	Y/Y	--	--	--/--
Patient 2	M	FXTAS	64/71	108	NA	Y/8	Y	N	20/ Dementia	Y	Y/N	Y	Y/Y	Y (caudate and putamen)/CC and Cereb
Patient 3	M	FXTAS*	61/63	92	NA	Y	N	ND	Y Cognitive decline	Y	N/N	NA	NA/NA	NA/NA
Patient 4	F	FXAND FXTAS	60/72	106	19	Y/5.5	Y	N	Y	N	N/N	N	Y/Y	N/Cereb
Patient 5	M	FXTAS	59/65	80	NA	Y/NA	Y	N	Dementia	N	Y/Y	Y	Y/Y	Y (Putamen)/ Supratentorial atrophy/Cereb
Patient 6	M	Asympt	NA/51	61	NA	N	N	N	N	N	N/N	N	N/N	NA/Cereb
Patient 7	F	Asympt	NA/76	62	19	N	N	N	N	N	N/N	N	Y/Y	N/ Cereb
Patient 8	M	FXAND FXTAS	72/81/*92*	74	NA	Y/3.5	Y	Y^α^	Y	Y	NA/Y	N	Y/N	Y (Pallidum and putamen)/CC and cereb
Patient 9	F	FXAND FXTAS*	44/46/*50*	82	23	Y/5.5	Y	Y^α^	Y	Y	N/Y	N	N/N	NA/Mild atrophy in cortex, cereb and mesencephalon
Patient 10	M	FXTAS	50/70	79	NA	Y/NA	Y	Y^α^	Y	N	N/N	N	Y/N	Y (Caudate and pallidum)/ Widespread severe atrophy, in CC and cereb
Patient 11	F	FXAND FXTAS*	55/55/*60*	90	30	Y/20.5	Y	N	Y	Y	Y/Y	N	Y/Y	N/Frontal atrophy
Patient 12	M	FXTAS	65/76//*86*	110	NA	Y	Y	Y^α^	Y	N	N/Y	N	Y/N	Y (caudate and putamen)/Central atrophy and cereb
Patient 13	M	FXAND FXTAS	49/49	82	NA	Y	Y	N	N	Y	N/N	--	--	--
Patient 14	F	Asympt	NA/42	106	30	N	N	N	N	N	N/N	--	--	--
Patient 15	F	FXAND	NA/34	89	33	N	N	N	Y	Y	NA	--	--	--
Patient 16	F	FXAND	NA/39	107	30	N	N	N	N	Y	N/N	N	Y/N	NA/N
Patient 17	F	Asympt	NA/29	135	36	N	N	N	N	N	N/N	NA	NA/NA	NA/NA
Patient 18	M	FXTAS	59/68/*84*	135	NA	Y	N	N	Y	N	Y/Y	N	Y/Y	NA/Widespread atrophy and cereb
Patient 19	M	Asympt	NA/79	84	NA	N	N	N	Y	N	NA	--	--	--/--
Patient 20	F	Asympt	NA/33	65	29	N	N	N	N	N	NA	NA	NA	NA/NA
Patient 21	F**	Asympt	40/62	77	32	N	N	N	N	N	NA	--	--/--	--/--
Patient 22	F	Asympt	NA/25	91	30	N	N	N	NA	N	NA	--	--/--	--/--
Patient 23	F	FXPOI	29/30	77	25	N	N	N	N	N	NA	--	--/--	--/--
Patient 24	F	FXPOI	39/51	86	20	N	N	N	N	N	NA	--	--/--	--/--
Patient 25	M	Asympt	NA/61	62	N	N	N	N	N	N	NA	N	Y/N	N/N
Patient 26	F	Asympt	NA/49	106	30	N	N	N	N	N	NA	--	--/--	--/--
Patient 27	F	Asympt	NA/51	114	29	N	Y	N	N	N	NA	N	Y/N	N/N
Patient 28	F	FXAND	NA/29	91	31	N	N	N	N	Y	N/N	NA	NA/NA	NA/NA
Patient 29	F	FXPOI	18/23	86	36	N	N	N	N	N	N/N	--	--/--	--/--
Patient 30	F	FXAND	NA/36	55	31	N	N	N	N	Y	N/N	NA	NA/NA	NA/NA
Patient 31	F	FXAND	NA/45	62	23	N	N	N	N	Y	N/N	N	N/N	N/Cereb
Patient 32	M	FXTAS	74/75	85	NA	Y	Y	Y	Y	N	YY	Y	Y/Y	Y (Caudate and pallidum)/Cereb
Patient 33	M	FXTAS	71/76	85	NA	Y	Y	Y	Y	N	Y/Y	N	Y/Y	Y (Pallidum)/Cereb

Age of onset for all FXTAS patients was 60.6 ± 8.9 years (range 44–74 years). Age of onset was significantly lower for women (53 ± 8.2) than for men (62.6 ± 8.2) (*p* = 0.048). Among those with FXTAS, 7 patients have neuropathy and 9 dysautonomia (Table 2). Mean CGG number for the entire cohort was 88.9 ± 20.1 repeats; mean CGG expansion in those with FXTAS was 94.3 ± 17.3 repeats, but the expansion size for men (94.3 ± 17.3 repeats) and women (92.7 ± 12.2 repeats) was not significantly different (Appendix A1, figure A1). Neither age of onset nor age of death correlated with CGG repeat expansion size in the complete FXTAS cohort; there was a strong correlation between CGG repeat numbers and SARA scores (*r* = 0.8) (Appendix A1, figure A2). A moderate inverse correlation was found between MoCA scores and CGG repeat expansion size (Appendix A1, figure A3).

Age of onset for FXPOI was 28.7 ± 10.5 years with GCC expansions ranging between 77 and 86 repeats. Age at menopause onset correlated with the healthy allele (*r*=-0.9) but not with the expanded one. There was not a significant difference in repeat numbers between those with FXTAS, FXPOI and FXAND when compared with asymptomatic PMC (data not shown). Delay to FXTAS diagnosis, calculated from symptom onset, for men was 7.3 ± 5.5 years, and for women 4.6 ± 6.4 years; for FXPOI diagnosis this delay was 6 ± 5.6 years (Appendix A1).

### Prediction of FXTAS Disease Status

A binary logistic regression was performed, examining prediction of FXTAS disease status. This model included gender, age, and number of CGG repeats, and significantly predicted disease status, χ² [3] = 18.91, *p* < 0.001. However, none of the individual predictors reached significance, though male gender showed a trend (*p* = 0.062).

### Prevalence

Among blood donors, with a total of 381 alleles analyzed, we did not find premutations (55–200 CGG) or intermediate alleles (45–54 CGG) in the *FMR1 gene* (Appendix A1 and figure A4).

## Neuroimaging

### Demographics and Radiologist’s Assessments

Demographics of the participants in the MRI analyses are shown in Table 3; no significant group differences in age and gender distribution were found. In the FXTAS group, deep and subcortical white matter lesions, cerebellar atrophy as well as parietal and temporal atrophy were found when compared to the control group. The MCP sign was present in 3/17 patients (2 men with FXTAS, 1 man with both FXTAS and FXAND) and the CCS sign in 8/17 (7 men with FXTAS and 1 asymptomatic woman). WMA, other than MCP and CCS signs, were present in 14/17 patients. Atrophy in the basal ganglia was found in 7/13 patients (for details, see Table 2). Variable signs of parkinsonism were present in 7 patients, of whom 5 had atrophy in the basal ganglia; in addition, 4 patients with parkinsonism had reduced dopamine transporter binding in the basal ganglia upon DaTSCAN whereas this examination was normal in a fifth FXTAS patient (Table 2). Various degrees of cerebellar atrophy were present in 13/17 patients whereas CC atrophy was in 3/17 patients (Tables 2 and 3). Some of the neuroradiological findings are shown in the supplementary document (Figure A5).

**Table 3:** Demographics of participants in the MRI analyses and results of radiologist’s assessments. ^§^ also called corpus callosum sign (CCS sign), asterisk denotes *p* < 0.05. Key: front = frontal; FXTAS = fragile X-associated tremor/ataxia syndrome; GCA = global cortical atrophy; MCP = middle cerebellar peduncle; NC-PMC = non-converted premutation carrier; occ = occipital; par = parietal; temp = temporal; WML = white matter lesions

Demographics	FXTAS	NC-PMC	Controls	*p*-value
Participants, N	10	8	20	
Age, Y (SD)	68.1 (10.6)	54.6 (13.1)	59.1 (13.6)	0.079
Gender, F/M	3/7	5/3	12/8	0.246
MCP sign	20%	12.50%	5%	0.442
Periventricular WML	90%	62.50%	45%	0.059
Deep WML	80%	25%	25%	0.009*
Subcortical WML	70%	12.50%	10%	0.001*
Splenium WML^§^	60%	25%	20%	0.077
Callosal WML (excludes splenium)	30%	12.50%	5%	0.161
Cerebellar atrophy (none/mild/severe)	2/4/4	4/3/1	9/11/0	0.041*
GCA front grade 0/1/2/3	3/3/4/0	5/2/1/0	13/6/1/0	0.143
GCA par 0/1/2/3	3/2/5/0	5/2/0/1	10/9/1/0	0.012*
GCA occ 0/1/2/3	8/1/1/0	7/1/0/0	20/0/0/0	0.250
GCA temp 0/1/2/3	2/8/0/0	5/3/0/0	18/3/0/0	0.001*

### Planimetric Measurements

For this analysis, 2 participants from the FXTAS group were excluded due to lack of appropriate T_1_-weighted sequence for planimetric measurements. For the FXTAS group there was a significant area reduction in the pons, midbrain and corpus callosum, see Table 4.

**Table 4:** Planimetric measurements. Asterisk (*) denotes *p* < 0.05 in one-way ANCOVA (controlled for age). Abbreviations: FXTAS = fragile X-associated tremor/ataxia syndrome; NC-PMC = non-converted premutation carrier

Manual segmentation	FXTAS (*N* = 8)	NC-PMC (*N* = 8)	Controls (*N* = 17)	*p*-value
Pons, mm3 (SD)	483.5 (49.0)	538.5 (48.0)	546.4 (63.6)	0.026*
Midbrain, mm3 (SD)	88.4 (22.7)	151.3 (34.0)	150.9 (23.7)	0.031*
Corpus callosum, mm3 (SD)	463.3 (69.1)	654.8 (105.0)	689.1 (95.7)	< 0.001*

### Volumetric Analysis

**Fig. 1:**
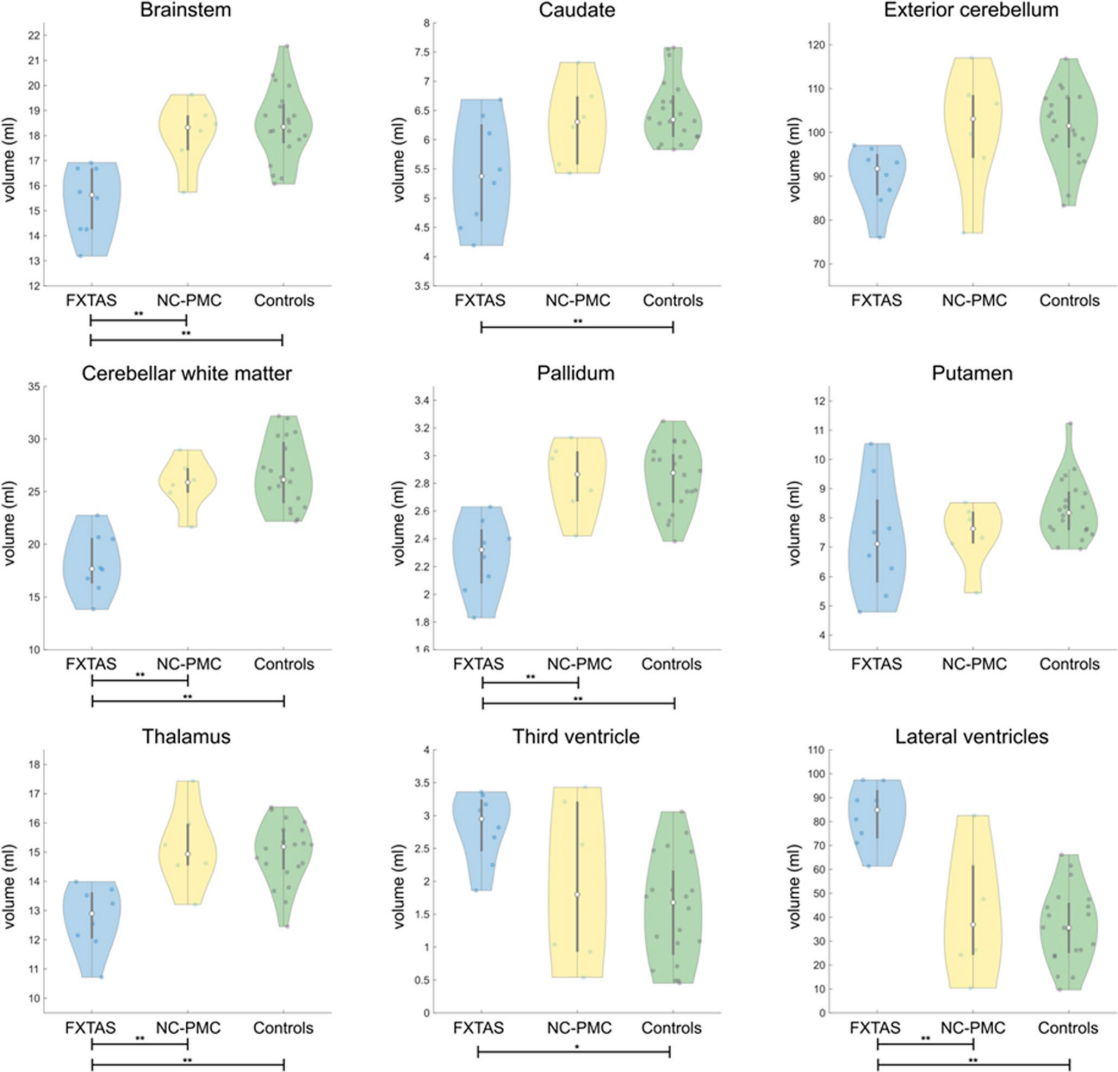
Violin plots of normalized volumetric measurements. Single asterisk (*) denotes *p* < 0.05 and double asterisks (**) denotes *p* < 0.01 in the pairwise comparisons. Key: FXTAS = fragile X-associated tremor/ataxia syndrome; NC-PMC = non-converted premutation carrier

Due to the lack of appropriate high-resolution T_1_-weighted sequence, an additional 2 participants from the non-converted premutation carriers (NC-PMC) group were excluded for this analysis, leaving a total of 8 FXTAS, 6 NC-PMC and 17 healthy controls. Significant differences in atrophy between the groups were found in the brainstem, caudate nucleus, cerebellar white matter, globi pallidi, thalamus. Enlargement of the third and lateral ventricles were also found. Results from group analysis of normalized volumes are given in Table 5 and visualized in Fig. 1.

**Table 5:** Results from volumetric analysis. Asterisk denotes p-value < 0.05. Abbreviations: FXTAS = fragile X-associated tremor/ataxia syndrome; NC-PMC = non-converted premutation carrier; WM = white matter

Automated volumetric segmentation	FXTAS (*N* = 8)	NC-PMC (*N* = 6)	Controls (*N* = 20)	ANCOVA *p*-value	FXTAS vs. PMC	FXTAS vs. Controls	PMC vs. Controls
Brainstem, ml (SD)	15.41 (1.37)	18.04 (1.34)	18.44 (1.44)	< 0.001*	0.007*	< 0.001*	0.503
Caudate, ml (SD)	5.42 (0.92)	6.28 (0.71)	6.49 (0.55)	0.026*	0.133	0.007*	0.393
Exterior cerebellum, ml (SD)	89.76 (7.05)	100.50 (13.87)	101.24 (8.44)	0.124			
Cerebellar WM, ml (SD)	18.22 (2.90)	25.74 (2.44)	26.69 (3.17)	< 0.001*	< 0.001*	< 0.001*	0.445
Pallidum, ml (SD)	2.27 (0.26)	2.83 (0.27)	2.84 (0.24)	< 0.001*	0.002*	< 0.001*	0.759
Putamen, ml (SD)	7.30 (1.98)	7.43 (1.10)	8.31 (1.06)	0.118			
Thalamus, ml (SD)	12.73 (1.10)	15.17 (1.43)	14.95 (1.08)	0.002*	0.003*	< 0.001*	0.810
Third ventricle, ml (SD)	2.82 (0.53)	1.95 (1.27)	1.57 (0.81)	0.019*	0.658	0.013*	0.055
Lateral ventricles, ml (SD)	82.59 (12.86)	42.08 (26.86)	36.30 (15.61)	< 0.001*	0.001*	< 0.001*	0.286

## Discussion

This is the first time a FXTAS cohort is reported in Scandinavia, and the presence of atrophy in the basal ganglia even if variable, in some patients is reported. Few others have reported atrophy in the basal ganglia, and thalamus [30, 31], and this atrophy was more pronounced among men with FXTAS than among asymptomatic PMC [30]. Larger studies are required to assess whether this atrophy correlates with clinical parkinsonism and/or reduced binding levels to the dopamine transporter. As in previous studies, the CCS sign was more common than the MCP sign [9, 16, 18]. We did not find a correlation between CGG expansion size and age of onset for ataxia, a similar lack of correlation was reported in a cohort of 50 men with FXTAS [8]. In contrast, other researchers have found significant correlations between age of onset and nucleotide expansion size [11, 12], reflecting phenotype heterogeneity in different populations. Penetrance in men in our cohort is higher than previously reported; male gender, age and CGG expansion size are important predictors for motor features and ultimately FXTAS penetrance [32, 33]. The prevalence of FXAND in our cohort is somehow lower than in previous studies [34, 35], but caution is required when interpreting these data since well-defined diagnostic criteria for FXAND are to be established. Autism spectrum disorder (ASD), ADHD, anxiety, and depression were reported as manifestations of FXAND among young PM patients [36]. In a recent review on FXAND, the most prevalent psychiatric disorders were neurodevelopmental disorders, anxiety, and bipolar disorder [34].

Our radiological findings are to be compared with other neurodegenerative diseases. For instance, the parkinsonism subtype of multiple system atrophy (MSA-P), but not the cerebellar form (MSA-C), is associated with basal ganglia atrophy [37]. Hyperkinetic disorders such as Huntington’s disease display progressive striatal atrophy predating clinical phenoconversion [38]. However, this atrophy is not specific for HD and occurs in neuroacanthocytosis syndromes as well [39].

A strength of this study is that all *FMR1* PMC were from a single center where longitudinal medical charts are available. However, this study is not without limitations, the main one being lack of in-depth cognitive assessments. Lack of wet biomarkers constitute another limitation as well as potential bias introduced by recruitment made at -a tertiary center. The disruption caused by the COVID19 pandemia explains why neuroimaging data was not done for all the patients. Also, those who completed neuroimaging studies were likely less severely affected than those who did not. Missing data may contribute to underestimating the presence of radiological FXTAS hallmarks. Furthermore, the small sample size limits exploration of genetic disease modifiers and larger correlations assessments. The significantly lower age of onset in women is reasonably explained by the combination of small sample size and the influence of the specific case of an aggressive course of disease in two deceased siblings. The lack of significant results regarding predictors for motor features and ultimately FXTAS penetrance in this study could also be seen in the light of a limited sample size.

Finally, it remains important to describe symptoms and signs to further understand both FXAND and the morbidity burden among PMC as well as to establish diagnostic criteria for FXAND.

## Conclusion

The clinical correlations of basal ganglia atrophy/ in FXTAS require larger cohort studies with long-term follow-up. However, our work adds to the growing literature on FXTAS and supports an appraisal to refine the current criteria for FXTAS.

## Supplementary Information

Below is the link to the electronic supplementary material.

Supplementary Material 1 (PDF 281 KB)

Supplementary Material 2 (PDF 163 KB)

Supplementary Material 3 (PDF 722 KB)

## Data Availability

Data is available from the corresponding author upon reasonable request.
