# Supplementary material for: Clinical Heterogeneity in a Scandinavian FMR1 Premutation Carrier Cohort and Basal Ganglia Atrophy in FXTAS
Source: Cerebellum. 2026 Feb 13;25(1):19. doi: 10.1007/s12311-026-01968-6 (PMC12904899; doi:10.1007/s12311-026-01968-6)
Supplement: Supplementary file 1 — Supplementary Material 1 (PDF 281 KB) [file 12311_2026_1968_MOESM1_ESM.pdf]

Supplementary document

# Clinical heterogeneity in a Scandinavian *FMR1* premutation carrier cohort and basal ganglia atrophy in FXTAS

## Patients and methods

The *FMR1* PMC in this study had been referred to the Department of Neurology or to the Department of Clinical Genetics and Genomics at the Karolinska University Hospital. They had mainly been identified through cascade testing, which occurs when a child is diagnosed with FXS (the full mutation carrier status). Cascade testing is offered to the expanded family to provide appropriate counselling, i.e for FXTAS. This is how the Department of Genetics at the Karolinska University Hospital in Stockholm has identified and diagnosed *FMR1* PMC. In two cases data collection was retrospective, since one patient was deceased at the time of data retrieval, and another one died during data collection. Medical records were extensively reviewed in all cases. Some patients declined to attend scheduled visits for neuroimaging studies and follow-up visits due to fear of contagion, advanced disease, claustrophobia and/or geographically inaccessibility. The following parameters were collected: age of onset, age at diagnosis, age at death, delay to diagnosis. The CGG repeat number in the expanded allele and for women also in the healthy allele were also collected.

## Prevalence study

A screening for *FMR1* premutations was carried out among healthy blood donors

## Statistics

All data are presented as mean ± standard deviation (SD), intergroup differences were assessed by t test, and Pearson correlation coefficients were calculated to establish the strength of association between variables. Significance level set at $p < .05$, all statistical analyses were performed using GraphPad Prism 9.

## Results

### Genetics

Blood donors went through genetic analyses using the following method.

PCR Amplification of the FMR1 CGG Repeat Region

The FMR1 CGG repeat region was amplified using a modified version of the protocol described by Almansour et al. (2022), employing Taq DNA Polymerase (Qiagen). Each 20 µL reaction contained 1× Qiagen PCR buffer, 0.6 mM dNTP mix, 0.1 mM 7-deaza-2′-deoxyguanosine (7-deaza-dGTP), 0.2 µM of each primer, 7.5% DMSO, 12.5% betaine, 0.5 U of Taq polymerase, and 50 ng of genomic DNA as template. The thermocycling profile consisted of an initial denaturation at 96 °C for 3 min, followed by 40 cycles of 96 °C for 30 s, 60 °C for 30 s, and 72 °C for 2 min, with a final extension at 72 °C for 5 min.

Two primer sets were used as reported by Almansour et al. (2022): (i) for repeat-primed PCR, forward 5′-TCAGGCGCTCAGCTCCGTTTCGGTTTCACTTCCGG-3′, reverse 5′-CCGGGAGCTGCATGTGTCAGAGGCCGCCGCCGCCGCCG-3′, and probe /56-FAM/CCGGGAGCTGCATGTGTCAGAGG-3′; and (ii) for conventional PCR, forward 5′-TCAGGCGCTCAGCTCCGTTTCGGTTTCACTTCCGG-3′ and reverse /56-FAM/AAGCGCCATTGGAGCCCCGCACTTCCACC-3′. PCR products were analyzed by capillary electrophoresis to determine fragment length and repeat pattern.

**Capillary Electrophoresis and Fragment Analysis**

PCR products were separated by capillary electrophoresis on an ABI 3730 Genetic Analyzer (Applied Biosystems) using GeneScan™ LIZ 500 as the internal size standard. Electrophoresis and data collection were performed under standard fragment analysis conditions according to the protocol reported by Almansour et al. (1). Electropherograms were analyzed using Peak Scanner 2 (Applied Biosystems) in overlay mode to visualize all samples simultaneously and confirm the reproducibility of fragment sizing across the dataset. The range of CGG expansion in the premutations and healthy alleles for *FMR1* in women is displayed in Table 2.

**Demographic data**

All patients but one (Pt 1) were evaluated at the Karolinska University Hospital (Table 2). Clinical notes, family history, and genetic tests were reviewed; phone interviews with caregivers were performed when

necessary. Age of onset did not correlate with CGG repeat expansion size in all the cohort or men alone but in women with FXTAS (r=0.9). These differences were reasonably explained by the small samples size for women (3 patients) and the presence of two striking outliers with aggressive and fatal course of disease in two sisters. There was a negative moderate correlation between CGG expansion size and cognitive performance in available MoCA score for 8 patients, 6 men and 2 women, diagnosed with FXTAS (r =-0.6). Age of death was 74.4 (5 observations) was 74.4±18.3, the correlations between age of death and CGG expansion size was weak (r=0.3). There was no correlation between age of death and CGG expansion size (data no shown).

Differences in delay to diagnosis between men and women with FXTAS was not significant (*t*-value is 0.35727, *p*-value is .363825); this delay was significantly different between those diagnosed with FXPOI and men with FXTAS (*t*-value =1.80701, *p*-value is .04908) but not with women diagnosed with FXTAS ( *t*-value is -0.27154. The *p*-value is .399708).

**CGG repeat and age of onset (full cohort)**

The scatter plot displays the relationship between *FMR1* CGG repeat size and age of onset in FXTAS among 14 individuals (including both males and females). Spearman's correlation analysis yielded a non-significant positive correlation (r = 0.1898, $p$ = 0.5115), with a wide 95% confidence interval ranging from –0.3938 to 0.6644. These findings indicate no statistically significant association between CGG repeat length and age of onset in this cohort.

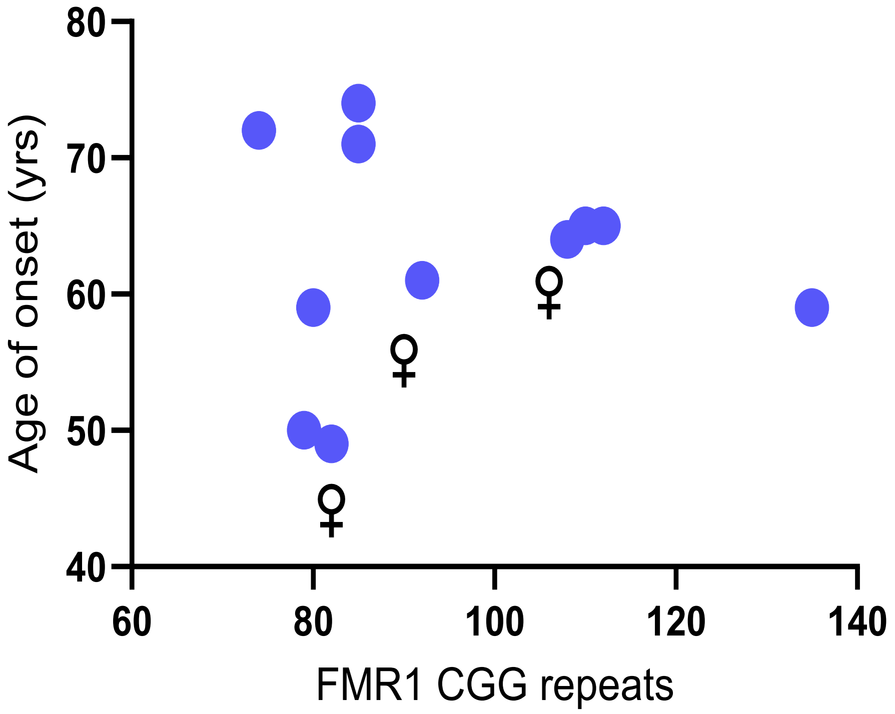


**Figure A1: Correlation between CGG repeat size and age of onset in FXTAS.** Scatter plot showing CGG repeat size versus age of onset (years) in 14 FXTAS cases. Females are marked with the ♀ symbol. No significant correlation was observed (Spearman's $r = 0.1898$, $p = 0.5115$), suggesting that CGG repeat length does not predict age of onset in this sample.

**Disease severity, and CGG repeat (SARA, full cohort)**

There was a significant positive correlation was observed between *FMR1* CGG repeat size and SARA score (a clinical measure of ataxia severity) in a group of 10 individuals, including both males and females. Spearman's correlation yielded r = 0.7908, with a p-value = 0.0084, indicating a statistically significant association. The 95% confidence interval ranged from 0.2401 to 0.9560. This suggests that higher CGG repeat lengths are associated with greater ataxia severity in this cohort.

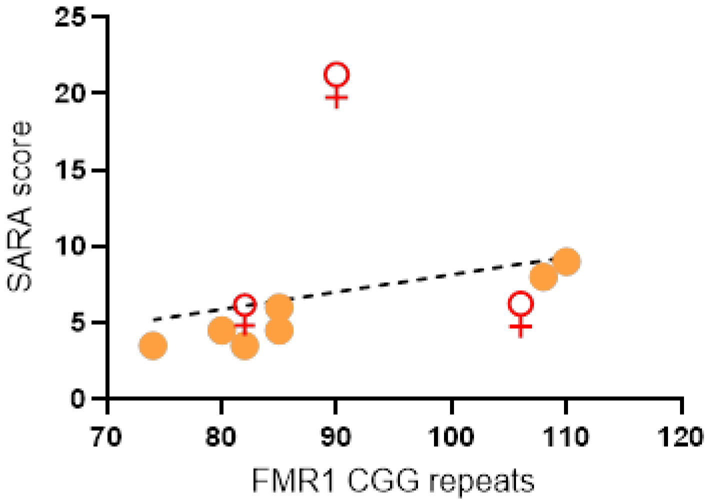


**Figure A2: Correlation between CGG repeat size and SARA score in *FMR1* premutation carriers.** Scatter plot depicting the relationship between FMR1 CGG repeat size and SARA score (Scale for the Assessment and Rating of Ataxia) in 10 individuals. Females are marked with the ♀ symbol. A strong, statistically significant correlation was found (Spearman's $r = 0.7908$, $p = 0.0084$), indicating that increased CGG repeat size is associated with degree of ataxia severity.

**Cognitive performance and CGG repeats**

A negative correlation was observed between *FMR1* CGG repeat size and MoCA in 8 individuals, including both sexes. Although the association did not reach statistical significance, Spearman's correlation yielded r = –0.7186, with a p-value = 0.0521, suggesting a trend toward lower cognitive performance with increasing CGG repeat size. The 95% confidence interval ranged from –0.9496 to 0.0466, reflecting variability due to small sample size.

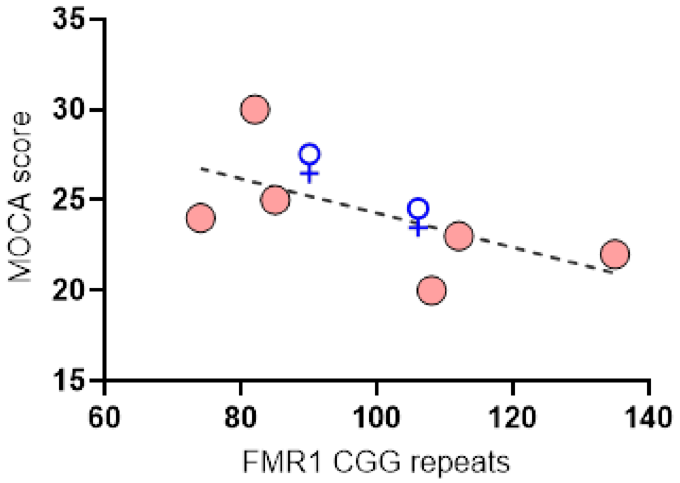


**Figure A3: Association between CGG repeat size and MoCA score in *FMR1* premutation carriers.** Scatter plot illustrating the relationship between CGG repeat length and MoCA (Montreal Cognitive Assessment) score in 8 participants. Females are indicated with the ♀ symbol. A negative trend was observed (Spearman's $r$ = –0.7186, $p$ = 0.0521), although not statistically significant. This trend may suggest that larger CGG expansions are associated with reduced cognitive performance.

**Missing data**

Missing imaging was due to limited mobility/advanced disease, geographically inaccessibility, claustrophobia and/or fear of coming to a hospital during the pandemic. In other cases (non-compatible software)/ older MRI studies with poor resolution and/or motion artifacts) precluded further analyses.

**Prevalence**

Among blood donors we did not find premutations or intermediate alleles in *FMR1* (45-54 CGG repeats).

**Repeat distribution in the Swedish population**

The histogram displays the distribution of *FMR1* CGG repeat sizes across 381 alleles. The distribution is unimodal and slightly right-skewed (skewness = 0.12), with a clear mode at 27 repeats, which represents the most frequent allele. A secondary peak is observed at 17 repeats. Repeat lengths range

from 14 to 42, with the interquartile range (IQR) between 21 and 27. The mean repeat size is 25.03 ± 5.33 (SD), and the median is 27 (95% CI: 26–27). The coefficient of variation is 21.28%, indicating moderate variability. The overall shape is leptokurtic (kurtosis = 0.56), with no extreme tails.

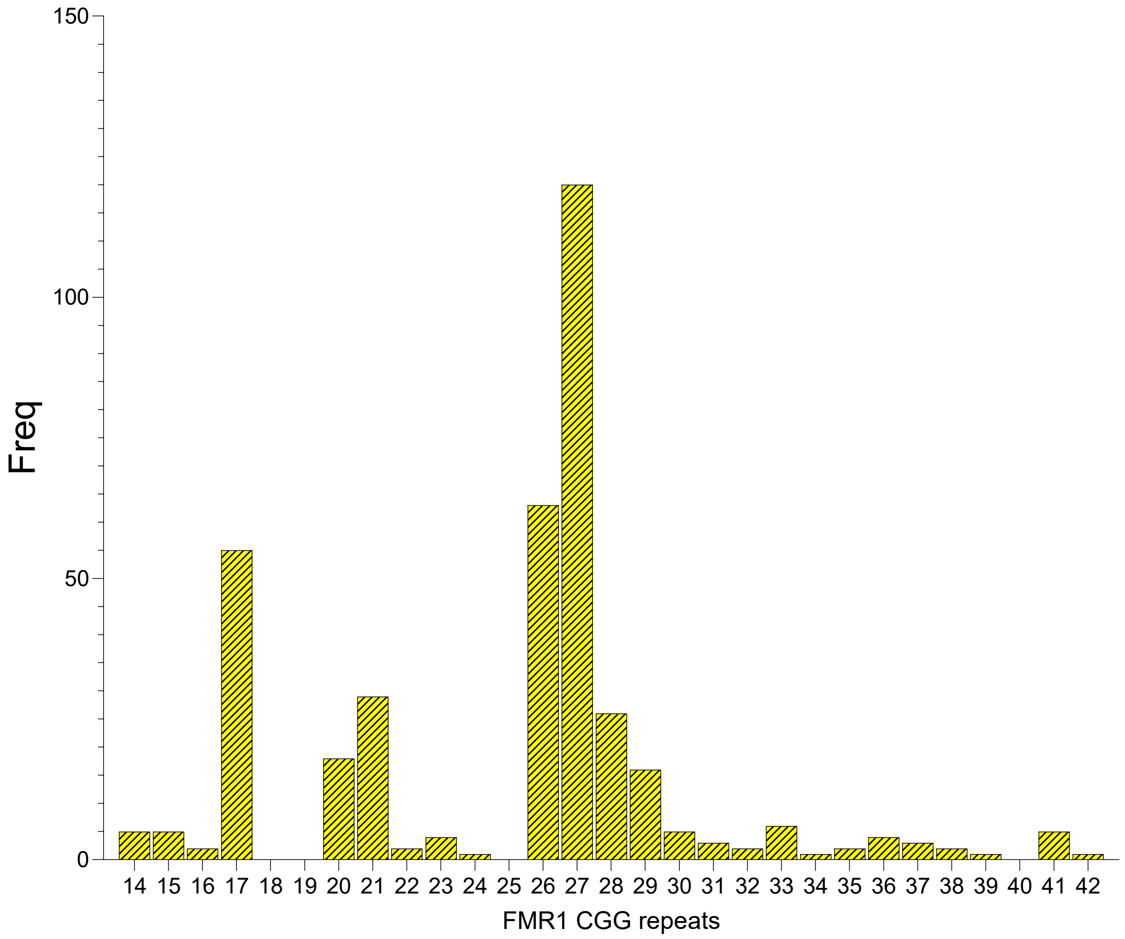


**Figure A4: Distribution of *FMR1* CGG repeat sizes in 381 alleles.** The histogram shows a unimodal distribution with a prominent peak at 27 CGG repeats. The mean repeat size was 25.03 (SD = 5.33), and the median was 27 (95% CI: 26–27). Repeat lengths ranged from 14 to 42. A secondary peak is evident at 17 repeats. The distribution showed slight positive skewness (0.12) and moderate kurtosis (0.56), indicating a slight right-tailed distribution without heavy outliers.

## Brain imaging

The controls for imaging studies were recruited among spouses/partners of patients with ALS. Imaging abnormalities among patients with FXTAS are summarized in table 2 and presented in figure A5.

## Psychiatric features

Psychiatric diagnoses based on DSM-IV criteria were as follows: recurrent depressive disorder in four patients (patients 4, 8, 16, and 31); combined anxiety and depressive episodes in four patients (patients 9, 11, 28, and 30); anxiety disorder in one patient (patient 13); attention-deficit/hyperactivity disorder in one patient (patient 28); bipolar disorder with comorbid personality disorder in one patient (patient 15); and obsessive–compulsive disorder in one patient (patient 30). Two patients had a history of substance abuse, and one patient had a history of anorexia. In patients with FXTAS, these psychiatric manifestations preceded the onset of motor symptoms. Available Hospital Anxiety and Depression

## References

1. Almansour, A., Ishiura, H., Mitsui, J. et al. Frequency of FMR1 Premutation Alleles in Patients with Undiagnosed Cerebellar Ataxia and Multiple System Atrophy in the Japanese Population. Cerebellum 21, 954–962 (2022). Available from: https://doi-org.proxy.kib.ki.se/10.1007/s12311-021-01329-
