# Supplementary material for: Clinical Heterogeneity in a Scandinavian FMR1 Premutation Carrier Cohort and Basal Ganglia Atrophy in FXTAS
Source: Cerebellum. 2026 Feb 13;25(1):19. doi: 10.1007/s12311-026-01968-6 (PMC12904899; doi:10.1007/s12311-026-01968-6)
Supplement: Supplementary file 2 — Supplementary Material 2 (PDF 163 KB) [file 12311_2026_1968_MOESM2_ESM.pdf]

Patients and parameters	Gender	Status/ diagnosis	Parkins.	Cognitive decline	Psych. features	Total HADS score/age of assessment	HADS-A	HADS-D	Neuropathy/ Dysautonomia
Patient 1	M	FXTAS	Y	23/ Dementia	N	NA*	NA	NA	Y/Y
Patient 2	M	FXTAS	N	20/ Dementia	Y	NA*	NA	NA	Y/N
Patient 3	M	FXTAS*	ND	Y Cognitive decline	Y	NA*	NA	NA	N/N
Patient 4	F	FXAND FXTAS	N	Y	N	16/73 y	10	6	N/N
Patient 5	M	FXTAS	N	Dementia	N	NA*	NA	NA	Y/Y
Patient 6	M	Asympt	N	N	N	0/51 y	0	0	N/N
Patient 7	F	Asympt	N	N	N	1/71 y	1	0	N/N
Patient 8	M	FXAND FXTAS	Y	Y	Y	NA*	NA	NA	NA/Y
Patient 9	F	FXAND FXTAS*	Y	Y	Y	NA*	NA	NA	N/Y
Patient 10	M	FXTAS	Y	Y	N	3/45 y	3	0	N/N
Patient 11	F	FXAND FXTAS*	N	Y	Y	NA	NA	NA	Y/Y
Patient 12	M	FXTAS	Y	Y	N	0/76 y	0	0	N/Y
Patient 13	M	FXAND FXTAS	N	N	Y	27/49 y	14	13	N/N
Patient 14	F	Asympt	N	N	N	0/53 y	0	0	N/N
Patient 15	F	FXAND	N	Y	Y	0/49 y	0	0	NA
Patient 16	F	FXAND	N	N	Y	6/45 y	5	1	N/N
Patient 17	F	Asympt	N	N	N	0/58 y	0	0	N/N
Patient 18	M	FXTAS	N	Y	N	NA*	NA	NA	Y/Y
Patient 19	M	Asympt	N	Y	N	0/79 y	0	0	NA
Patient 20	F	Asympt	N	N	N	3/33	3	0	NA
Patient 21	F	Asympt	N	N	N	7/63 y	4	3	NA
Patient 22	F	Asympt	N	NA	N	5/25 y	2	3	NA
Patient 23	F	FXPOI	N	N	N	10/31 y	7	3	NA
Patient 24	F	FXPOI	N	N	N	0/ 51 y	0	0	NA
Patient 25	M	Asympt	N	N	N	5/62 y	5	0	NA
Patient 26	F	Asympt	N	N	N	NA	NA	NA	NA
Patient 27	F	Asympt	N	N	N	6/52 y	4	2	NA
Patient 28	F	FXAND	N	N	Y	27/30 y	15	12	N/N
Patient 29	F	FXPOI	N	N	N	14/24 y	9	5	N/N
Patient 30	F	FXAND	N	N	Y	15/37 y	13	2	N/N
Patient 31	F	FXAND	N	N	N	6/48 y	4	2	N/N
Patient 32	M	FXTAS	Y	Y	N	4/75 y	2	2	YY
Patient 33	M	FXTAS	Y	Y	N	6/77 y	4	2	Y/Y

Table A1: Distribution of The Hospital Anxiety and Depression Scale (**HADS**) scores in a *FMR1* cohort at a tertiary center in Sweden. *NA: not applicable due to frank dementia. Key: asympt: asymptomatic; A: anxiety, D: depressive symptoms. Pt 9 suffered from anxiety and depression many years before onset of PSP and FXTAS.
