# Supplementary material for: Clinical Heterogeneity in a Scandinavian FMR1 Premutation Carrier Cohort and Basal Ganglia Atrophy in FXTAS
Source: Cerebellum. 2026 Feb 13;25(1):19. doi: 10.1007/s12311-026-01968-6 (PMC12904899; doi:10.1007/s12311-026-01968-6)
Supplement: Supplementary file 3 — Supplementary Material 3 (PDF 722 KB) [file 12311_2026_1968_MOESM3_ESM.pdf]

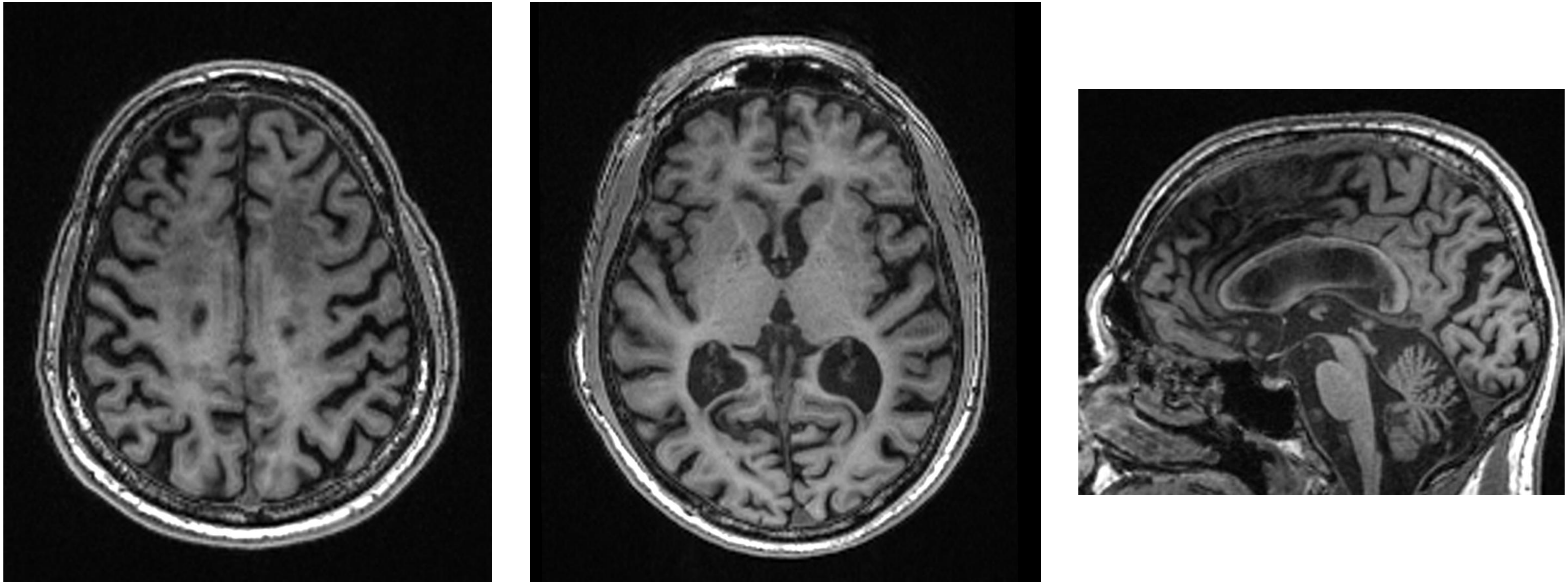

A B C

Patient 5

Male with probable FXTAS. T1 weighted MRI sections displaying atrophy in the cortex (A), the putamen (B) and cerebellum (C) .

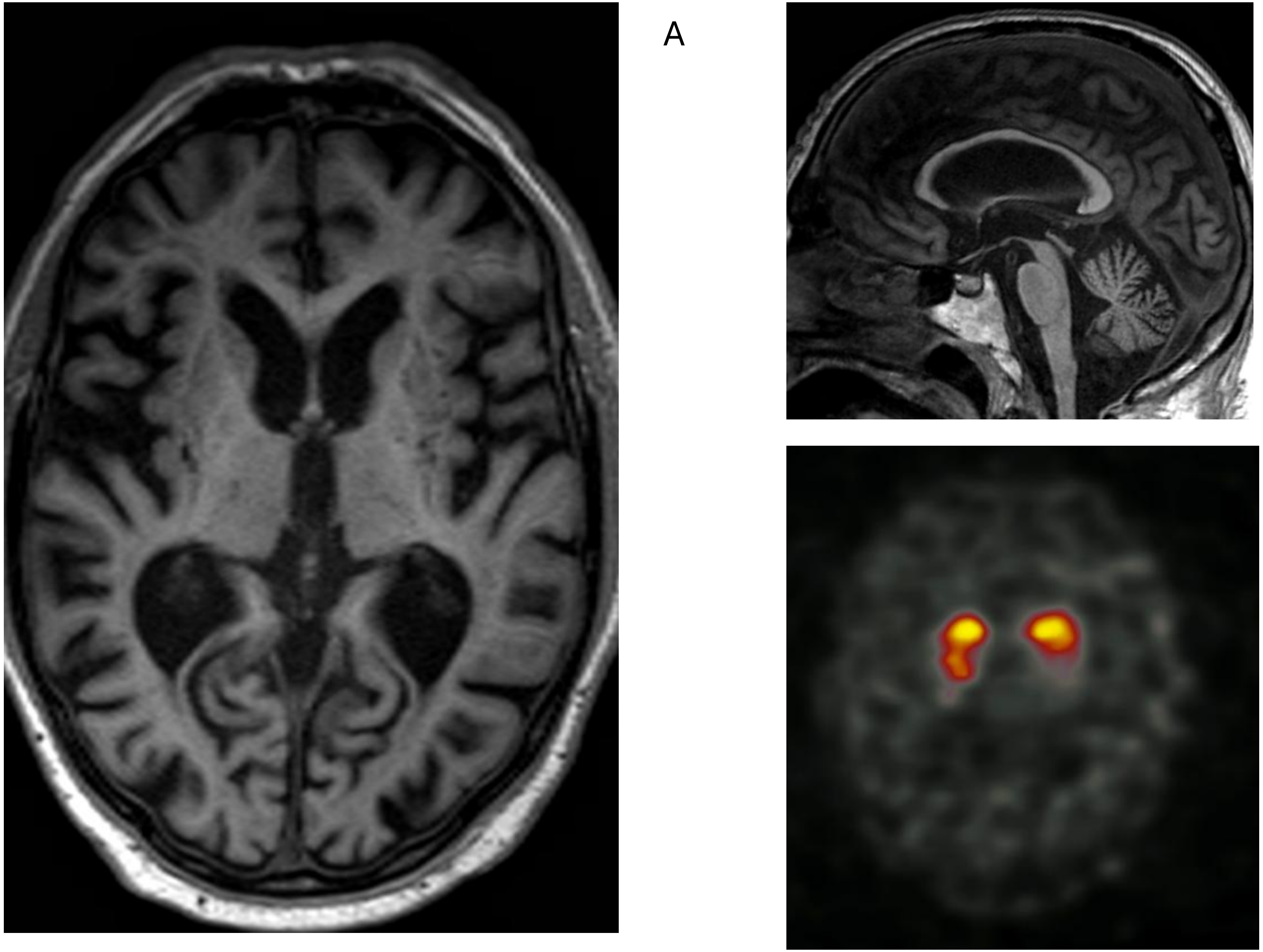

Patient 10
Male with definitie FXTAS. T1 weighted MRI sequences showing:
A) Atrophy in the putamen, caudate and pallidum.
B) Atrophy in the corpus callosum and in the cerebellum.
C) DaTSCAN (BRASS, Hermes Medical Solutions): Bilateral reduced uptake in putamen more pronounced on the left side as well as in caudate nuclei.

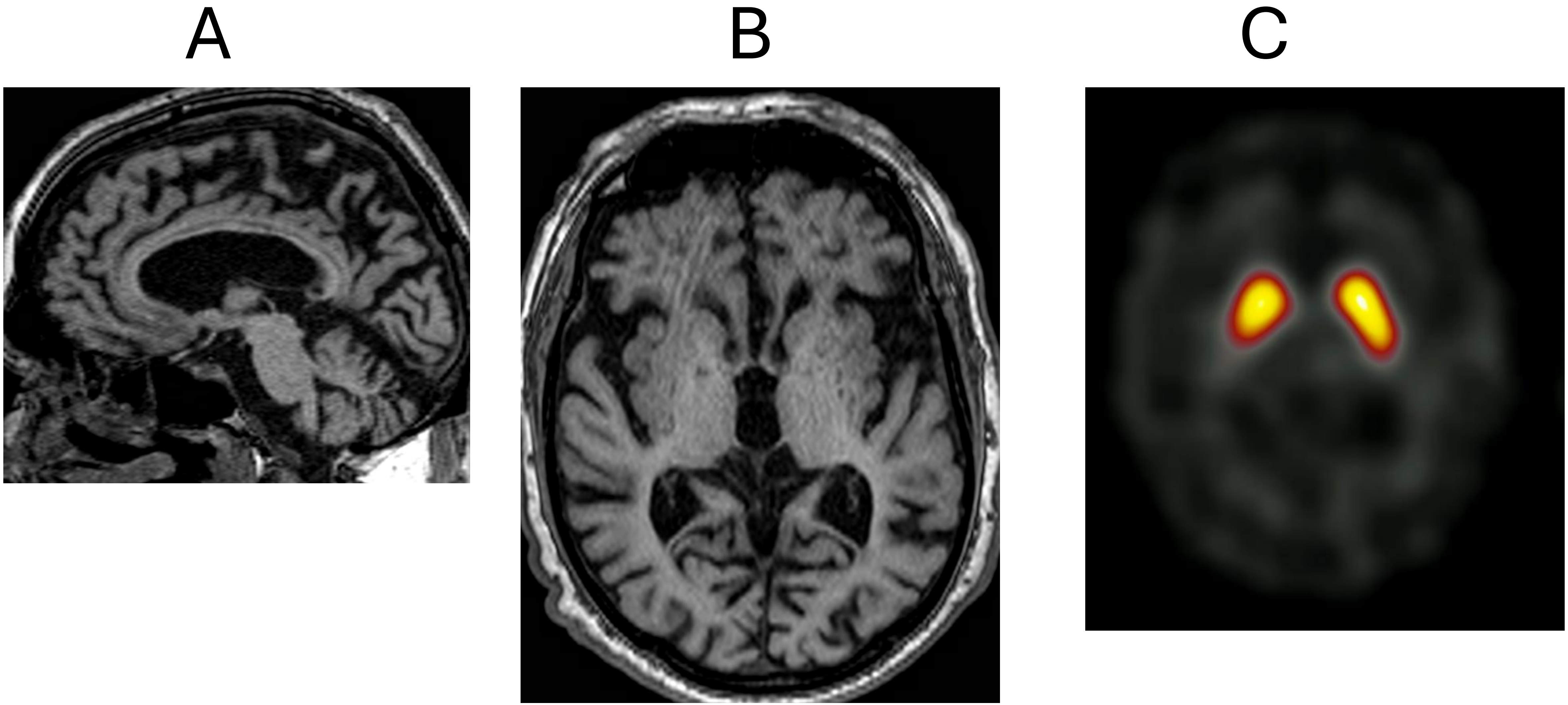

Patient 12

Male with probable FXTAS. T1-weighted MRI sequences showing: cerebellar atrophy (A) and atrophy in the caudate and putamen. DaTSCAN : Asymmetrical reduction of the uptake in the right putamen.

A

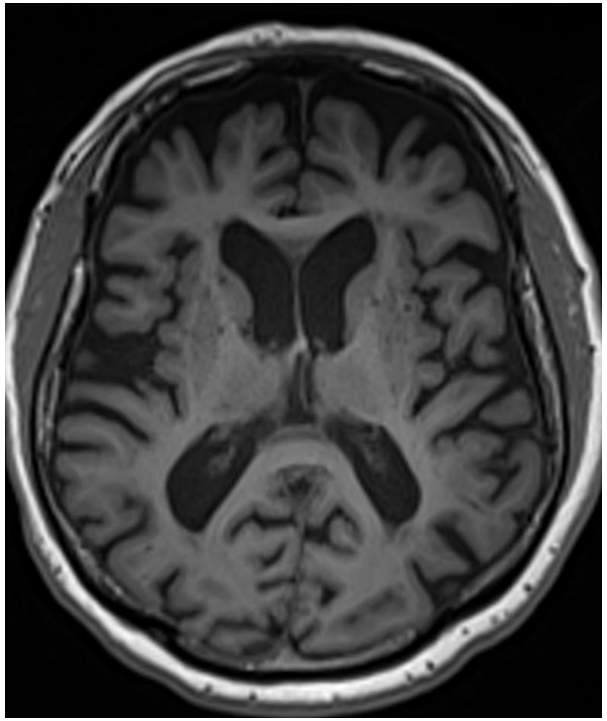

B

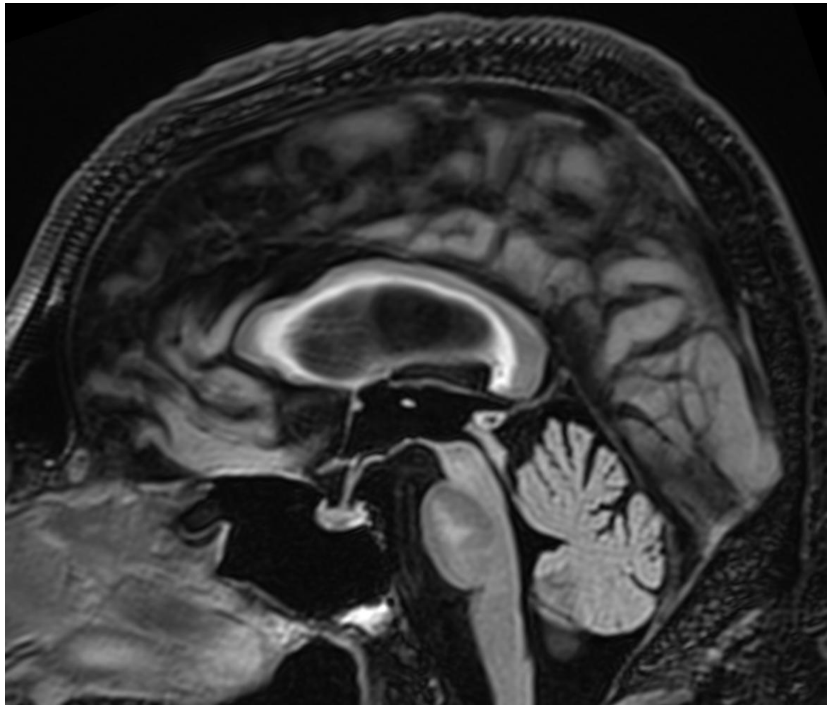

C

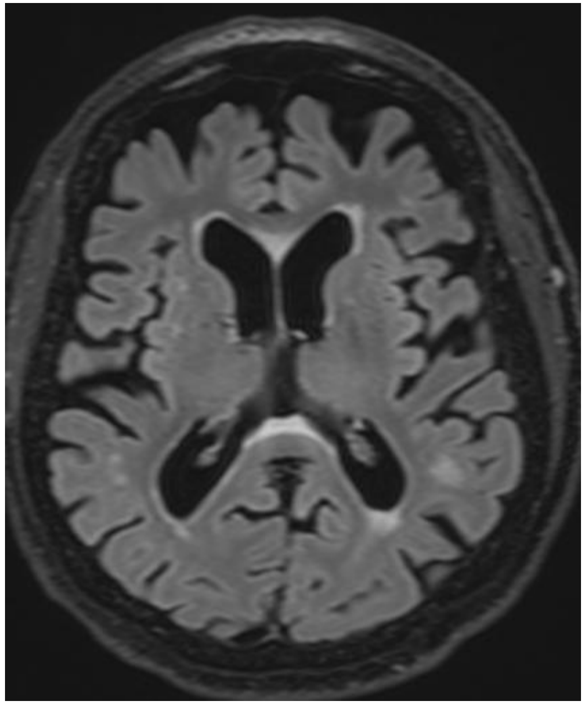

Patient 32
Male with definite FXTAS. T2 FLAIR images showing atrophy is in both the caudate nuclei and pallidum (A), cerebellum (B) and corpus callosum (CC) hyperintensities (B and C).

A B C

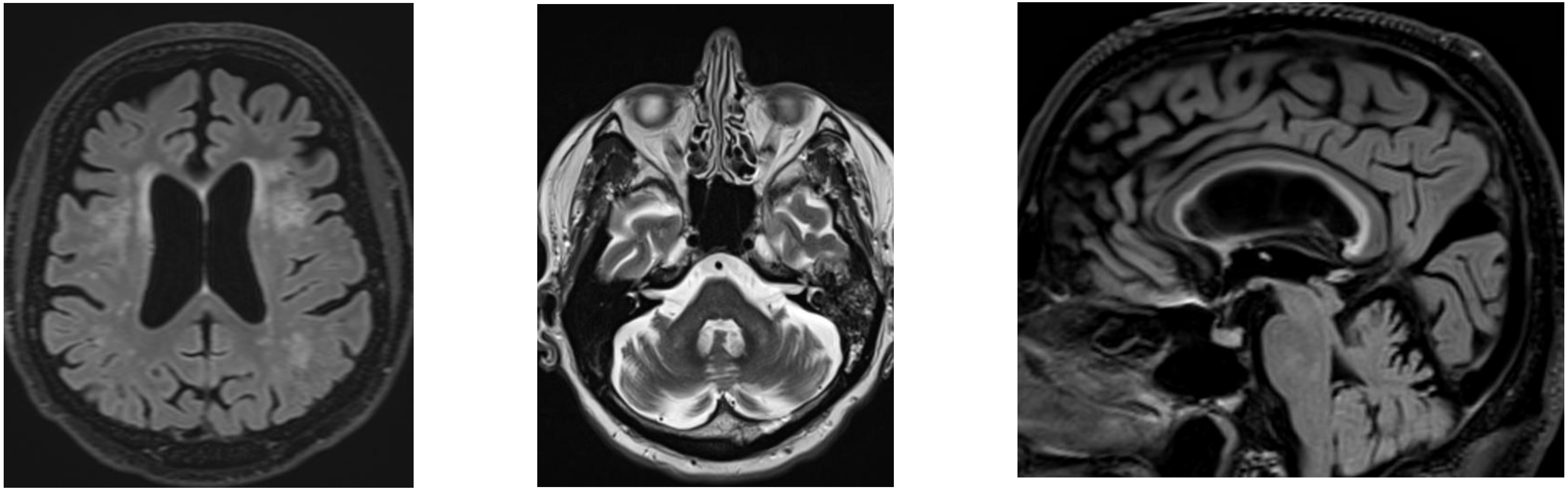

Patient 32

T2 FLAIR image shows periventricular hyperintensities (A), T2 weighted TSE shows hyperintensities in the middle cerebellar peduncle (MCP sign) (B) are shown

Patient 33

T2 FLAIR image in this man with probable FXTAS shows CC hyperintensities and cerebellar atrophy (C).
